# Specific Resting-State Brain Networks in Mesial Temporal Lobe Epilepsy

**DOI:** 10.3389/fneur.2014.00127

**Published:** 2014-07-14

**Authors:** Mona Maneshi, Shahabeddin Vahdat, Firas Fahoum, Christophe Grova, Jean Gotman

**Affiliations:** ^1^Montreal Neurological Institute and Hospital, McGill University, Montreal, QC, Canada; ^2^Functional Neuroimaging Unit, Centre de Recherche de l’Institut Universitaire de Gériatrie de Montréal, Montreal, QC, Canada; ^3^Multimodal Functional Imaging Laboratory, Department of Biomedical Engineering, McGill University, Montreal, QC, Canada

**Keywords:** temporal lobe epilepsy, independent component analysis, resting-state fMRI, brain networks, functional connectivity

## Abstract

We studied with functional magnetic resonance imaging (fMRI) differences in resting-state networks between patients with mesial temporal lobe epilepsy (MTLE) and healthy subjects. To avoid any *a priori* hypothesis, we use a data-driven analysis assessing differences between groups independently of structures involved. Shared and specific independent component analysis (SSICA) is an exploratory method based on independent component analysis, which performs between-group network comparison. It extracts and classifies components (networks) in those common between groups and those specific to one group. Resting fMRI data were collected from 10 healthy subjects and 10 MTLE patients. SSICA was applied multiple times with altered initializations and different numbers of specific components. This resulted in many components specific to patients and to controls. Spatial clustering identified the reliable resting-state networks among all specific components in each group. For each reliable specific network, power spectrum analysis was performed on reconstructed time-series to estimate connectivity in each group and differences between groups. Two reliable networks, corresponding to statistically significant clusters robustly detected with clustering were labeled as specific to MTLE and one as specific to the control group. The most reliable MTLE network included hippocampus and amygdala bilaterally. The other MTLE network included the postcentral gyri and temporal poles. The control-specific network included bilateral precuneus, anterior cingulate, thalamus, and parahippocampal gyrus. Results indicated that the two MTLE networks show increased connectivity in patients, whereas the control-specific network shows decreased connectivity in patients. Our findings complement results from seed-based connectivity analysis (1). The pattern of changes in connectivity between mesial temporal lobe structures and other areas may help us understand the cognitive impairments often reported in patients with MTLE.

## Introduction

Mesial temporal lobe epilepsy (MTLE) is a common form of human focal epilepsy, with hippocampal sclerosis a common underlying pathology (2). Although seizures in MTLE heavily involve the temporal lobes, it is now clear that there are more anatomically widespread functional disturbances (3). In addition, it appears that structural and metabolic abnormalities in this population are not limited to the period of seizure occurrence and probably affect the periods with no epileptic discharges, i.e., the resting-state periods.

A method to investigate how different parts of the brain interact with each other is to measure the intrinsic function of the brain at resting-state using functional magnetic resonance imaging (fMRI). Because this approach does not require subjects to perform a specific task, it is attractive for clinical studies. In MTLE, studies have shown changes in functional connectivity of the temporal or mesial structures with other brain areas (1, 4–6), and reported impaired resting-state networks including the perceptual, attention, and default mode networks (DMN) (1, 7–10). Studies combining fMRI resting-state functional connectivity and diffusion tensor imaging (7, 11) suggest that functional connectivity changes in MTLE are affected by loss in gray matter volume and white matter integrity in the temporal lobe.

Independent component analysis (ICA) is a popular method to analyze resting-state fMRI data since it provides a network view of the changes in brain activity, by decomposing the data into statistical independent spatial components, each component being associated with a time-course (12). The main limitation of ICA is its nature, which does not generalize simply to drawing conclusions about groups of subjects. Despite this issue, a number of group-ICA approaches have been proposed (13, 14). These approaches differ in terms of data organization prior to the ICA analysis, types of available output, and the statistical approaches. However, there are challenges concerning most of the available group-ICA approaches, extensively discussed by Vahdat et al. (15), which cause ambiguity in the classification and detection of components at the group level.

We recently proposed a new ICA-based method to address the limitations of the current ICA approaches in the situation of multi-groups/multi-conditions comparisons (15). The method, called “shared and specific independent component analysis” or “SSICA,” systematically performs between-group network comparisons. It extracts and classifies components (networks) into two categories: those that are common to groups and those that are specific to one of the groups. This is done by adding a constraint to the FastICA (16) algorithm to simultaneously deal with the data of multiple groups within one ICA estimation.

Here we studied, using fMRI, group-specific differences in resting-state networks between patients with unilateral MTLE and healthy control subjects. For this purpose, we considered the SSICA, since it does not require any *a priori* hypothesis, and therefore, can assess differences between the groups independently of the involved structures. Moreover, we were interested in finding the most reliable resting-state networks among all the components that are extracted as specific, and also in estimating functional connectivity in each group and exploring its changes across groups.

## Materials and Methods

### Subjects

Ten patients with unilateral MTLE (aged 29 ± 11 years, 3 males, 7 right MTLE) were selected from our EEG–fMRI dataset of patients scanned at 3-T. These patients were a subset whose fMRI data fulfilled our criteria for selecting patients, as explained below. All patients were taking medication at the time of study and they did not stop it for the purpose of scanning. The study was approved by the research ethics board of the Montreal Neurological Institute and Hospital and subjects participated in the research after giving written informed consent. Patients’ inclusion criteria were: (a) having a unilateral mesial temporal epileptic focus according to the clinical information (history of febrile seizures, seizure types, and EEG and MRI findings), (b) no large structural or postsurgical lesion in order to ensure the accurate coregistration with the average standard space, (c) having at least two fMRI runs with no interictal epileptic discharges (IEDs) proven by the simultaneous EEG recording, (d) wakefulness proven by EEG recording during these runs, and (e) motion of <1 mm as determined by the realignment of the preprocessing (see “fMRI Preprocessing,” preprocessing step 5). Table 1 gives the demographic and clinical characteristics of all patients. Ten healthy controls (aged 32 ± 9 years, 5 males) were scanned using the same fMRI protocol, fulfilling inclusion criteria (d) and (e). There was no significant difference between the age distributions of the two groups (sign test, *p* > 0.05). Subjects were asked to keep their eyes closed during the scan and were instructed to refrain from any structured thoughts and from falling asleep.

**Table 1:** **Patients’ clinical data**.

Patient	Gender	Age/ onset of epilepsy	Epilepsy type	History of febrile seizures	Seizure types	Interictal EEG	Ictal EEG	MRI	Anti- epileptic medications
1	F	20/15	R MTLE	No	Psychic aura, LOC, and postictal fatigue	R T spikes ≫ L T spikes	N/A	R hippocampal and parahippocampal lesion	VPA, LEV, and PB
2	F	36/7	R MTLE	Yes	Epigastric aura, LOC, oral automatism, and R hand automatism	R T spikes	R T	R HA	CBZ
3	M	29/14	L MTLE	No	Aura of déjà vu, LOC, oral automatism, and rare GTCS	L T spikes and L T slow waves	L T	Non-lesional	CBZ
4	F	46/32	L MTLE	Yes	Olfactory aura, L hand automatism, R hand dystonia, and postictal dysphasia	L T spikes ≫ R T spikes	N/A	L HA and HS	TPM and OXC
5	M	18/17	R MTLE	No	Aura of déjà vu, LOC, and rare GTCS	L T spikes and L T slow waves	N/A	R T DNET and R HA	GBP and CLB
6	F	40/39	R MTLE	Yes	Olfactory aura, sensation of coldness, bad odor, LOC, and oral automatisms	R T spikes	R T	Non-lesional	CBZ and CLB
7	F	27/6	R MTLE	Yes	Epigastric aura, warm sensation, fear, tachycardia, postictal confusion, and rare GTCS	R T spikes and sharp waves	R T	R mesial temporal atrophy	CBZ and CLB
8	M	19/14	L MTLE	No	Epigastric aura, LOC, L hand automatism, and R hand dystonia	L T spikes	N/A	L HA and HS	CBZ, CLB, and LTG
9	F	16/5	R MTLE	Yes	Aura of déjà vu, LOC, and manual automatism	R T spikes and R T rhythmic slow waves	N/A	R HA and HS	VPA, CLB, LEV, and TPM
10	F	40/1	R MTLE	Yes	Epigastric aura, nausea, and LOC	R T spikes and R T slow waves	N/A	R HA and HS	VPA, LEV, and PB

*MTLE, mesial temporal lobe epilepsy; R, right; L, left; T, temporal; M, male; F, female; HA, hippocampal atrophy per MRI; HS, hippocampal hyperintensity per MRI; DNET, dysembryoplastic neuroepithelial tumor; VPA, valproate; CLB, clobazam; LEV, levetiracetam; CBZ, carbamazepine; LTG, lamotrigine; GBP, gabapentin; TPM, topiramate, OXC, oxcarbazepine, PB, phenobarbital; LOC, loss of consciousness; GTCS, generalized tonic clonic seizures*.

### EEG acquisition

The EEG acquisition was performed using 25 MR compatible electrodes (Ag/AgCl) placed on the scalp using the 10–20 (21 usual electrodes without Fpz and Oz, reference at FCz) and 10–10 (F9, T9, P9, F10, T10, and P10) electrode placement system, as described elsewhere (17). Two electrodes located on the back recorded the electrocardiogram (ECG). To minimize movement artifacts and for the patient’s comfort, the head was immobilized using a pillow filled with foam microspheres (Siemens, Germany). Data were transmitted from a BrainAmp amplifier (Brain Products, Munich, Germany, 5 kHz sampling rate) via an optic fiber cable to the EEG monitoring computer located outside the scanner room.

### fMRI acquisition

Functional images were continuously acquired using a 3-T MR scanner (Siemens Trio, Germany). A T1-weighted anatomical acquisition was first done (1 mm slice thickness, 256 × 256 matrix, TE = 9.2 ms, TR = 22 ms, and flip angle 30°). Four to seven fMRI runs, each recording 200 volumes, were acquired for each patient and 2–4 runs for each healthy control. TLE patients selected for this study had at least two runs with no epileptic discharges seen on EEG. In order to have the same number of runs for all subjects, only two runs for every patient and control subject were selected. For patients, fMRI data were collected with two EPI acquisition protocols: (I) 5 scans done before July 2008: voxel dimensions 5 mm × 5 mm × 5mm, 25 slices, 64 × 64 matrix, TE = 30 ms, TR = 1750 ms, and flip angle 90°, (II) 5 scans after July 2008: voxel dimensions 3.7 mm × 3.7 mm × 3.7 mm, 33 slices, 64 × 64 matrix, TE = 25 ms, TR = 1900 ms, and flip angle 70°. All the healthy controls were scanned with protocol (II).

### EEG processing

The brain vision analyzer software (Brain Products, Munich, Germany) was used for off-line correction of the gradient artifact and filtering of the EEG signal. This software uses the method described by Allen and colleagues (18). A 50-Hz low-pass filter was also applied to remove remaining high-frequency artifact. The ballistocardiogram (BCG) artifact was removed by ICA (19, 20). A neurologist reviewed the EEG recordings and made sure that the selected runs in patients were free of epileptic discharges and that the patients and controls were awake during these runs.

### fMRI preprocessing

Regular preprocessing was performed using FMRIB software library (FSL), www.fmrib.ox.ac.uk, Oxford, UK, FSL version 4.1 (21, 22). The following preprocessing steps were applied: (1) flipping of patients’ data to make a homogeneous left MTLE group (10 cases) and increase the sample size, (2) removal of the first two volumes of each scan to allow for equilibrium magnetization, (3) slice timing correction using Fourier-space time-series phase-shifting, (4) non-brain tissue removal (23), (5) motion correction using a six-parameter linear transformation using a maximization of the correlation ratio (default settings of FSL) (24), (6) intensity normalization of all volumes of each run as implemented in FSL (7) spatial smoothing using a Gaussian kernel with 6 mm full width at half maximum (FWHM), and (8) high-pass temporal filtering with cut off frequency of 0.01 Hz. To achieve the transformation between the low-resolution functional data and the average standard space [MNI152: average T1 brain image constructed from 152 normal subjects (25)], we performed two transformations. The first was from the low-resolution EPI image to the T1-weighted structural image (using 7 degrees of freedom affine transformation), and the second was from T1-weighted structural image to the average standard space (using a 12 degrees of freedom linear affine transformation, voxel size = 2 mm × 2 mm × 2 mm). Data were then sub-sampled to 4 mm isotropic space to limit the computational burden.

### SSICA method

**Figure 1:**
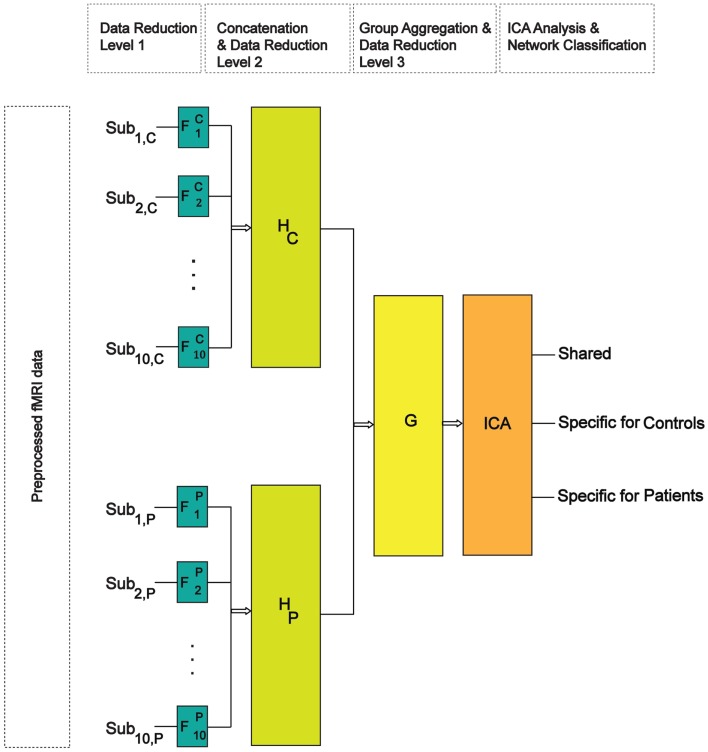
**Schematic of the SSICA algorithm**. There are three levels of data whitening and reduction. F, H, and G, respectively represent the transformation matrices at the first (subject), second (within-group), and the third (between-group) levels of data reduction.

Shared and specific independent component analysis employs a three-step data reduction and whitening procedure prior to its simultaneous ICA analysis and network-classification (see Supplementary Material; Figure 1 for details). Here, the size of each subject’s preprocessed data was reduced from 2 × 198 to 50 time-points by performing the first principal component analysis (PCA). We chose 50 time-points since it explained at least 90% of data variability in each subject’s dataset. In total 30 components (shared and specific together) were extracted in both groups. This was done on the aggregate reduced data of both groups, where the size of each group’s concatenated data was reduced from (10 × 50) to (30-*K*2) for group-1 and (30-*K*1) for group-2; where *K*1 and *K*2 are the maximum number of specific components in group-1 and group-2. In order to test the stability of our results with respect to the total number of extracted components, we did additional analyses by extracting 40 and 50 components at the group level.

Shared and specific independent component analysis was applied several times with different numbers of extracted specific components. We chose to extract up to three specific components per group [*K*1 and *K*2 = (0, 1, 2, and 3)], as allowing more specific components only resulted in repetition or combination of already-extracted specific components (i.e., it did not introduce any new component). Therefore, we considered four possible values for the number of specific components, and for each run of the SSICA, we assigned one of these values to the number of extracted specific components in each group (e.g., 0 for group-1 and 1 for group-2, 0 for group-1 and 2 for group-2, 0 for group-1 and 3 for group-2, and so on). Consequently, the number of possible combinations with repetition was 4 × 4, equal to 16. Excluding the condition where 0 specific networks are extracted in both groups, we ended up with 15 cases. We repeated the whole procedure five times to account for the effect of the ICA initialization, which introduces stochastic behaviors of ICA algorithms and could play an important role in algorithmic instability (26). So in total 15 × 5 = 75 SSICA estimations were considered for further analysis. The number of extracted specific components for patients with MTLE was 118, while for controls 39 specific components were extracted. It should be noted that the outputs of SSICA are spatial *Z*-score maps.

### Spatial clustering analysis

The remaining important issue was to find the most reliable specific components among all the specific components in each group. To do so, the following analysis based on the clustering method proposed by Hyvarinen and Ramkumar (27) was performed. In their method, the null hypothesis models the case where the components for different subjects/sessions have no similarity at all, other than what would be expected by chance (randomness). They introduced a null distribution, which embodies the two possible sources of such randomness (scenario I. complete failure of the ICA algorithm and scenario II. the underlying components are completely different for each subject). For constructing the null distribution, instead of using an explicit model of multivariate distribution, they proposed a model where parameters can be directly estimated from the observed data. Using the *p*-values computed for the similarities between sets of components, they proposed a hierarchical clustering procedure, where median was used as the linkage strategy and determined the pairwise distance between components. Correcting for multiple testing, this clustering method controls the false positive rate (FPR) for the formation of clusters, and the false discovery rate (FDR) for adding new elements to clusters.

As implemented in the clustering package ISCTEST, for each group, our input to the clustering algorithm was a three-dimensional tensor containing *k* 2-D matrices (*k* being the total number of specific components extracted in that group), where each 2-D matrix was an extracted specific component’s spatial map. We set the FPR and FDR to the conservative value of 10% as recommended by Hyvarinen and Ramkumar (27) in the context of real fMRI. The outputs of this analysis were several clusters of specific components, which had the highest within-cluster similarities and the lowest between-clusters similarities (simple spatial correlation was used as the similarity metric). Then, all the components within each cluster were averaged to obtain a representation of that cluster. Results were then overlaid on standard MNI152 at 1 mm resolution for visualization purposes. The thresholded *Z*-maps (*Z* > 2.3) were labeled according to the Harvard–Oxford cortical and subcortical (28), and Juelich histological atlases (29).

### Functional connectivity estimation

In this part of the analysis, we were interested to estimate the functional connectivity of each reliable specific resting-state network and to compare it between groups. Here, we defined functional connectivity of a network based on the power of its corresponding time-course in the frequency band of the resting-state BOLD signal (0.01–0.1 Hz). For each reliable specific network and each subject (patient or control), we used the subject’s fMRI data and the network’s spatial map in a general linear model (GLM) to find one associated time-course per network and subject. We then used power spectrum analysis (with the standard Hamming window as implemented in MATLAB) to assess the power of this estimated time-course within the 0.01–0.1 Hz frequency band. For each group and each reliable specific network, power was averaged across subjects within that group to calculate the functional connectivity of that network.

## Results

Following the clustering algorithm described above, two significant clusters of components were detected specific to the MTLE group and one specific to the control group. These clusters respectively included 111, 6, and 23 specific components (in total there were 118 specific components in the MTLE and 39 in the control group). Setting FPR and FDR thresholds of the clustering analysis at 10%, 1 component in patients and 16 in controls were not included into any significant cluster. The three reliable specific networks, corresponding to these three clusters, are illustrated in Figures 2A–C. As explained before, all the components within each cluster were averaged to obtain the representation of that cluster (the reliable specific network).

**Figure 3:**
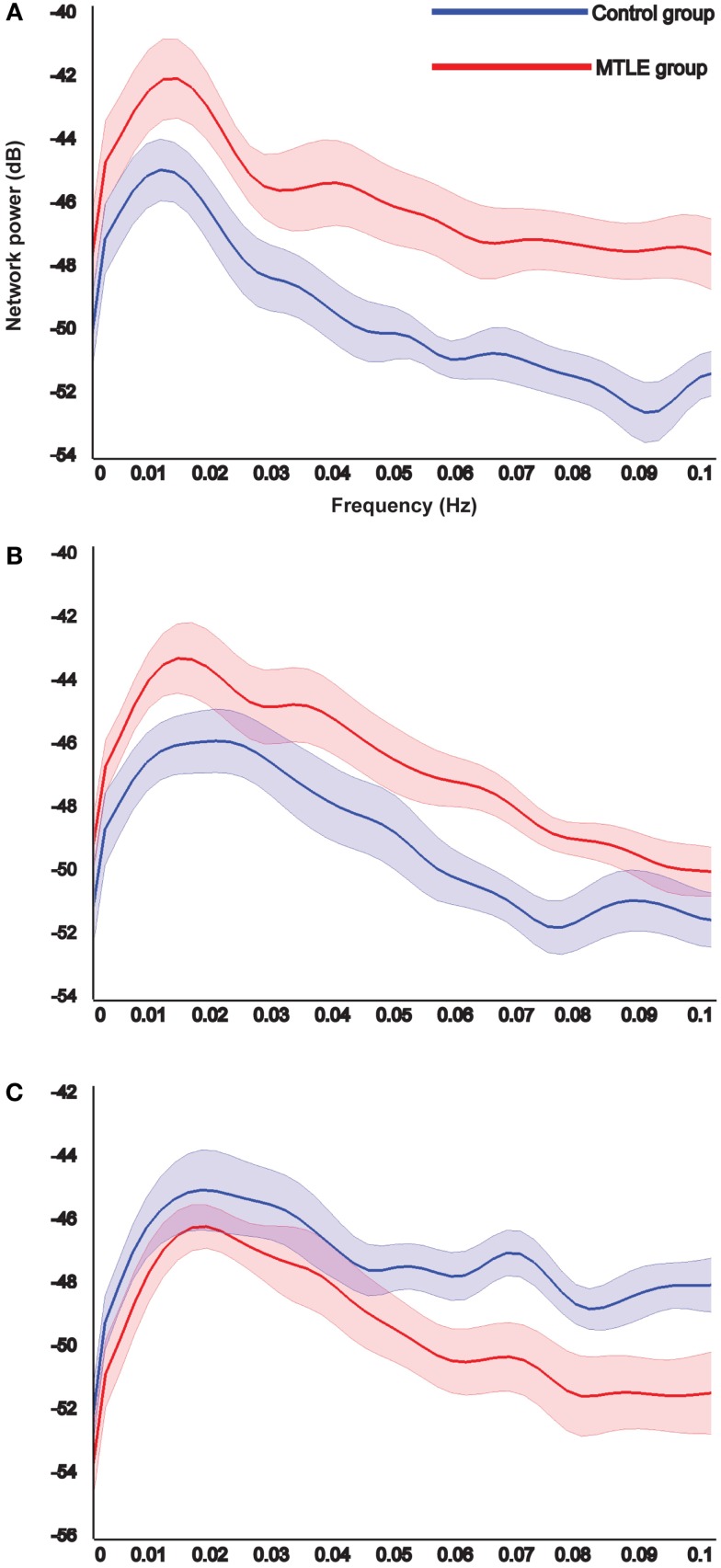
**Results of the power spectrum analysis on the temporal dynamics of each detected specific resting-state networks**. The reliable resting-state networks specific to the MTLE group show increased functional connectivity in patients compared to controls **(A,B)**, whereas the reliable resting-state network specific to the controls shows the opposite **(C)**. *X*-axis shows the frequency in Hertz and *Y*-axis indicates the power in decibel. Shaded area shows standard error of the mean.

**Figure 2:** **The three detected reliable specific resting-state networks**. Reliable resting-state networks specific to the MTLE group **(A,B)**, reliable resting-state networks specific to the control group **(C)**. Note that this result is showing the average of spatial maps within each reliable cluster. *Z*-values range between 2.3 and 5 in both cases. To be compatible with the results in Figure 3, we chose to illustrate the MTLE-specific networks **(A,B)** in red and the control-specific network **(C)** in blue.

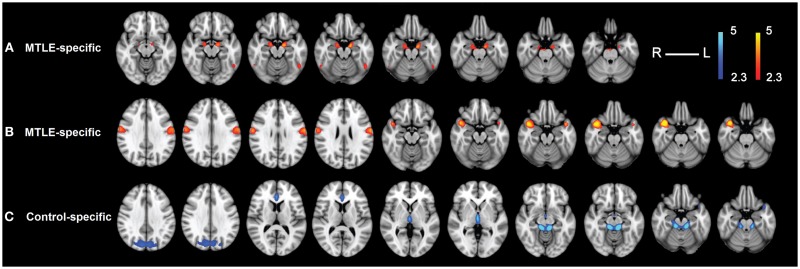



Our result in Figure 2A demonstrates that the most reliable MTLE-specific network comprises bilateral hippocampi, amygdalae, and inferior temporal gyri (more on the side of focus). The other reliable MTLE-specific network comprises the postcentral gyri and bilateral temporal pole, with more involvement on the healthy side (Figure 2B). Results in Figure 2C demonstrate that the reliable control-specific network, comprising precuneus, anterior cingulate, thalamus, brainstem, and parahippocampal gyrus. For the cases where 40 or 50 components were extracted at the group level, we found very similar results as when 30 components were extracted.

Results of power spectrum analysis on the temporal dynamics of the detected resting-state networks show that the two MTLE-specific networks show increased functional connectivity in the patients compared to the controls (Figures 3A,B), whereas the control-specific network shows decreased functional connectivity in patients (Figure 3C). This explains why we chose to illustrate the MTLE-specific networks (Figures 2A,B) in red and the control-specific network (Figure 2C) in blue.

## Discussion

We used an ICA-based analysis to study resting-state brain activity in patients with MTLE and investigated the resting-state networks specific to them when compared to healthy controls. Following the framework proposed in SSICA, we assume that the specific networks are those that differ when comparing both groups; either a normal network identified in controls, which is less or more present in patients, or a pathological network that only exists in the patients. The SSICA requires as input the number of networks specific to each group but the true value of this number is not known *a priori*. We therefore ran the SSICA multiple times, with different maximum numbers of specific components, providing us with a large number of components specific to the patients and to the controls (we also ran SSICA with multiple initializations to increase statistical performance and decrease sensitivity to initial conditions). A subsequent clustering analysis on the specific components estimated in each group resulted in the detection of two reliable resting-state networks specific to the MTLE group and one specific to the control group. To explore changes of functional connectivity across groups, power spectrum analysis was performed. This analysis demonstrated that the two reliable MTLE-specific networks show increased functional connectivity in the MTLE group compared to the healthy control group, whereas the one control-specific network shows the opposite.

In a previous seed-based functional connectivity study by our group, Pittau et al. (1) demonstrated that amygdala and hippocampus on the affected and to a lesser extent, on the healthy side are functionally less connected with contralateral homologous structure. Our results demonstrated that the most reliable MTLE-specific network includes bilateral hippocampus and amygdala, more on the side of the focus and comprises regions where functional connectivity, measured through power of network, is higher in patients than in controls.

Even though at first sight this result may seem to be contradiction with the finding of Pittau’s study, we believe that this difference is originating from dissimilar strategies for functional connectivity estimation. The difference can be explained as follows: one may assume that as a result of sporadic epileptic discharges, the BOLD signal extracted from the seeds in the affected areas, specifically the hippocampus and amygdala, show more variability compared to the signal from the same areas in healthy controls. Since in the context of SSICA functional connectivity of a resting-state network is defined based on the power of its corresponding time course, this extra variability of the BOLD signal within the MTLE group could result in its extraction as a specific network. However, as a result of the same variability, the correlation between the BOLD signals extracted from the affected and the healthy hippocampus and amygdala could be reduced and therefore, decreased functional connectivity will be detected using seed-based analysis.

Bettus et al. also reported complementary but inconsistent information on functional connectivity in TLE measured by BOLD signal and by intracerebral EEG (iEEG). In their study, using both modalities, functional connectivity was estimated during the interictal period in epileptic and in non-affected regions. Functional connectivity measured from the iEEG signal was reported to be higher in affected regions compared to non-affected areas, whereas an opposite pattern was found for functional connectivity measured from the BOLD signal (4). Using regional homogeneity (ReHo) as an index of ongoing activity, Zeng et al. also reported increased synchronized brain activity, in MTLE patients relative to controls, in some regions including ipsilateral parahippocampal gyrus (30).

Our other finding was that the second most reliable MTLE-specific network shows increased functional connectivity in patients compared to controls between the postcentral gyri and bilateral temporal poles. As also suggested by Maccotta et al. (31), we believe that as a result of recurrent seizures, structural degeneration and decreased connection density, or a combination of both, some neural connections may be facilitated, which in turn lead to elevation of functional connectivity within MTLE-specific networks. In this regard, Holmes et al. investigated the gray matter concentration in left TLE at the voxel level and found decrease in patients in a network comprised of left hippocampus and left postcentral gyrus (32). In a quantitative MRI study, Coste et al. (33) demonstrated that in refractory TLE, the temporal pole is frequently atrophic ipsilateral to seizure onset. Labate et al. (34), using cortical thickness for assessment of neuropathologic changes, demonstrated progressive neocortical atrophy in intractable MTLE patients, which likely represents seizure-induced damage. The involvement of neocortical regions, such as sensorimotor cortex, in the pathophysiology of TLE has also been reported by other authors (35, 36).

Finally, we found that the reliable control-specific network, comprised of precuneus, anterior cingulate, thalamus, brainstem, and parahippocampal gyrus, shows decreased functional connectivity in patients compared to controls. We find this result consistent and complementary to the findings of Pittau et al. (1), which demonstrated that in MTLE patients compared to controls, amygdala and hippocampus on the affected and to a lesser extent on the contralateral side are functionally less connected with the dopaminergic mesolimbic network and the DMN. We believe that in MTLE, the amount of correlation between the BOLD signals extracted from seeds in the affected areas and remote regions will be reduced since distant regions do not necessarily show BOLD changes related to epileptic discharges. Moreover, as the BOLD signals extracted from regions beyond the affected structures in TLE do not necessarily have more variation in patients compared to controls, the control-specific network shows less power in patients compared to controls. In a recent study by McCormick et al. (37), patients with MTLE showed reduced resting-state functional connectivity from the posterior cingulate cortex to the epileptogenic hippocampus. Zeng et al. (30) also reported decreased ReHo in DMN, including precuneus and posterior cingulate gyrus, bilateral inferior lateral parietal and mesial prefrontal cortex. In addition, Liao et al. (7) showed that in MTLE patients compared to controls, functional and structural connectivity of the bilateral mesial temporal lobes were significantly decreased with the posterior cingulate cortex and with precuneus and suggested that in MTLE, the decreased connection density in several areas in the DMN might be responsible for decreased functional connectivity within this network.

It is important to note that a causal relationship cannot be inferred from the current analysis and our results simply reflect the state of the brain of patients with MTLE, which may relate to structural abnormalities, long-standing epilepsy, or medication, a combination of these, or a common cause for this type of epilepsy. The fact that patients were on different medications may be considered as a confounding factor between patients and controls in our analysis. Unfortunately, it is almost impossible to dissociate the long term effect of medication from the effect of disease when studying patients with a long duration of epilepsy since the vast majority of patients take a combination of different medications since the onset of their disease.

Given the number of patients, this study did not allow us to investigate the correlation between the functional connectivity of the two detected reliable MTLE-specific resting-state networks and the duration of epilepsy. However, it would be interesting for future studies to investigate those networks that show greater alterations in functional connectivity in patients with a longer history of disease. In addition, given the small number of subjects in each group of MTLE patients, we could not study the two groups separately and therefore were not able to investigate whether there are different mechanisms underlying left and right MTLE.

We want to reemphasize that although SSICA and seed-based functional connectivity analysis measure different aspects of brain activity organization and sometimes give apparently inconsistent results, they may complement each other and provide more information about the underlying processes resulting in changes of functional connectivity.

## Conflict of Interest Statement

The authors declare that the research was conducted in the absence of any commercial or financial relationships that could be construed as a potential conflict of interest.

## Supplementary Material

The Supplementary Material for this article can be found online at http://www.frontiersin.org/Journal/10.3389/fneur.2014.00127/abstract
