# Supplementary material for: Specific Resting-State Brain Networks in Mesial Temporal Lobe Epilepsy
Source: Front Neurol. 2014 Jul 14;5:127. doi: 10.3389/fneur.2014.00127 (PMC4095676; doi:10.3389/fneur.2014.00127)
Supplement: Supplementary file 1 [file Presentation_1.PDF]

Appendix: SSICA Model

In ICA framework, a T×M matrix of random variables Y is decomposed based on the following generative model:

$$Y = AS \qquad (1)$$

Where S is an N×M matrix whose rows are mutually independent (N < T) (sources), and A is a T×N mixing matrix. Each row of matrix S and its corresponding column in the mixing matrix constitute a single component. In the context of fMRI connectivity analysis, T is the number of acquired volumes in time, M is the number of voxels, and sources are the brain networks.

Let n_1 be the number of subjects in group-1, and n_2 the number of subjects in group-2, and assume Y_i^j denotes the zero-mean data of subject i in group j ($Y_i^j$is a T^j_i×M dimension matrix where T^j_i is the number of acquired volumes for that subject and M is the number of voxels after co-registration).

In SSICA, the first level PCA is applied using the projection matrix, F_i^j. Although this first data reduction step is not necessary, it is recommended in fMRI analysis due to the computational burden.

$$X_i^j = F_i^j \, Y_i^j \qquad (2)$$

Temporal concatenation of data of different subjects in one single matrix is done using the following equation, where $\tilde{X}_1$ and $\tilde{X}_2$ are the concatenated reduced data of group-1 and group-2, respectively.

$$\tilde{X}_1 = \begin{bmatrix} X_1^1 \\ . \\ . \\ . \\ X_{n_1}^1 \end{bmatrix};\ \tilde{X}_2 = \begin{bmatrix} X_1^2 \\ . \\ . \\ . \\ X_{n_2}^2 \end{bmatrix} \qquad (3)$$

At the second level PCA, the temporally concatenated data of all subjects of each group is whitened using the following equation:

$$X_j = H^j\ \tilde{X}_j,\ j = 1{,}2 \qquad (4)$$

Where H^1 is an $\mathrm{N}_{g1} \times n_1\mathrm{T}_1$ whitening matrix for group-1, and H^2 is an $\mathrm{N}_{g2} \times n_2\mathrm{T}_2$ whitening matrix for group-2. Therefore, X_1 and X_2 denote the whitened concatenated observed data of group-1 and group-2, respectively.

At the third level PCA, group data are aggregated row-wise, and further whitened and reduced using a $\mathrm{N} \times (\mathrm{N}_{g1} + \mathrm{N}_{g2})$ projection matrix, G, where N defines the resulting number of independent components that will be extracted by ICA.

$$X = G \begin{bmatrix} X_1 \\ X_2 \end{bmatrix} \qquad (5)$$

Let us assume that the true number of components in group-1 and group-2 are respectively $\mathrm{K_{g1}}$ and $\mathrm{K_{g2}}$ (therefore, data can be reconstructed by $\mathrm{K_{g1}}$ and $\mathrm{K_{g2}}$ independent components). In SSICA framework, we decompose the generative model given in Equation 1 into two parts for each group: components shared between the two groups ($\boldsymbol{s}_i^{sh}$), and components specific to each group ($\boldsymbol{s}_i^{sp,1}$or $\boldsymbol{s}_i^{sp,2}$).

$$\begin{bmatrix} X_1 \\ X_2 \end{bmatrix} = \begin{bmatrix} A_1^{sh} & A_1^{sp} & 0 \\ A_2^{sh} & 0 & A_2^{sp} \end{bmatrix} \begin{bmatrix} S^{sh} \\ S_1^{sp} \\ S_2^{sp} \end{bmatrix} \qquad (5)$$

Where K is the number of shared components among them ($\boldsymbol{s}_i^{sh}, i = 1,..,\mathrm{K}$), $\boldsymbol{s}^{sh}$ and $\boldsymbol{s}^{sp,j}$ are columns of $S_j{}^T,\ j = 1,2$ arranged according to the shared and specific labeling, and $\boldsymbol{a}^{sh,j}$**,** $\boldsymbol{a}^{sp,j}$ are the corresponding columns in the mixing matrices A_j^{sh} and A_j^{sp}. Here, $\mathrm{K}_1 = \mathrm{K}_{\mathrm{g1}} - \mathrm{K}$ is the true number of specific components of group-1 ($\boldsymbol{s}_i^{sp,1}, i = 1,..,\mathrm{K}_1$), and $\mathrm{K}_2 = \mathrm{K}_{\mathrm{g2}} - \mathrm{K}$ is the true number of specific components of group-2 ($\boldsymbol{s}_i^{sp,2}, i = 1,..,\mathrm{K}_2$). Note that, the reduced dimension of aggregate data specified at the third level PCA, N, should be set greater than or equal to the total number of components in both groups (i.e. $\mathrm{K}_1 + \mathrm{K}_2 + \mathrm{K}$). Also, in SSICA the maximum number of specific components that can be extracted is $\mathrm{N} - \mathrm{N}_{g2}$ for group-1, and $\mathrm{N} - \mathrm{N}_{g1}$ for group-2, hence $\mathrm{N}, \mathrm{N}_{g1}$, and N_{g2} should be set such that $\mathrm{N} - \mathrm{N}_{g2} \geq \mathrm{K}_1$ and $\mathrm{N} - \mathrm{N}_{g1} \geq \mathrm{K}_2$.

Equation 5, the independent component factorization, can be integrated into the FastICA cost function (Hyvarinen, 1999) using the Lagrange multipliers method (Lang, 1987), and then be optimized using Newton's method. This gives the SSICA iterative formula, Equation 2.19 in Vahdat et al. (2012), which allows to simultaneously maximize the independency at the component level and the specific components orthogonality at the group level. For further details on solving these equations, see Vahdat et al. (2012).
